# Comparison of the pathogenesis of SARS-CoV-2 infection in K18-hACE2 mouse and Syrian golden hamster models

**DOI:** 10.1242/dmm.049632

**Published:** 2022-11-11

**Authors:** Haengdueng Jeong, Youn Woo Lee, In Ho Park, Hyuna Noh, Sung-Hee Kim, Jiseon Kim, Donghun Jeon, Hui Jeong Jang, Jooyeon Oh, Dain On, Chanyang Uhm, Kyungrae Cho, Heeju Oh, Suhyeon Yoon, Jung Seon Seo, Jeong Jin Kim, Sang-Hyuk Seok, Yu Jin Lee, Seung-Min Hong, Se-Hee An, Seo Yeon Kim, Young Been Kim, Ji-Yeon Hwang, Hyo-Jung Lee, Hong Bin Kim, Dae Gwin Jeong, Daesub Song, Manki Song, Man-Seong Park, Kang-Seuk Choi, Jun Won Park, Jun-Young Seo, Jun-Won Yun, Jeon-Soo Shin, Ho-Young Lee, Ki Taek Nam, Je Kyung Seong

**Affiliations:** ^1^ Severance Biomedical Science Institute, Brain Korea 21 FOUR Project for Medical Science, Yonsei University College of Medicine, Seoul 03722, South Korea; ^2^ Department of Nuclear Medicine, Seoul National University Bundang Hospital, Seongnam 13488, South Korea; ^3^ Institute of Immunology and Immunological Diseases, Yonsei University College of Medicine, Seoul 03722, South Korea; ^4^ Korea Mouse Phenotyping Center, Seoul National University, Seoul 08826, South Korea; ^5^ Department of Microbiology, Yonsei University College of Medicine, Seoul 03722, South Korea; ^6^ Laboratory of Developmental Biology and Genomics, Research Institute for Veterinary Science, and Brain Korea 21 FOUR Program for Creative Veterinary Science Research, College of Veterinary Medicine, Seoul National University, Seoul 08826, South Korea; ^7^ Division of Biomedical Convergence, College of Biomedical Science, Kangwon National University, Chuncheon 24341, South Korea; ^8^ Laboratory of Avian Diseases, Brain Korea 21 FOUR Program for Veterinary Science and Research Institute for Veterinary Science, College of Veterinary Medicine, Seoul National University, Seoul 08826, South Korea; ^9^ Preclinical Research Center, Seoul National University Bundang Hospital, Seongnam 13488, South Korea; ^10^ Department of Periodontology, Section of Dentistry, Seoul National University Bundang Hospital, Seongnam 13620, South Korea; ^11^ Department of Internal Medicine, Seoul National University Bundang Hospital, Seoul National University College of Medicine, Seongnam 13620, South Korea; ^12^ Bionanotechnology Research Center, Korea Research Institute of Bioscience and Biotechnology, Daejeon 34141, South Korea; ^13^ Department of Veterinary Medicine Virology Laboratory, College of Veterinary Medicine and Research Institute for Veterinary Science, Seoul National University, Seoul 08826, South Korea; ^14^ Science Unit, International Vaccine Institute, Seoul 08826, South Korea; ^15^ Department of Microbiology, Institute for Viral Diseases, Biosafety Center, Korea University College of Medicine, Seoul 02841, South Korea; ^16^ Laboratory of Veterinary Toxicology, College of Veterinary Medicine, Seoul National University, Seoul 08826, South Korea; ^17^ Department of Nuclear Medicine, Seoul National University, College of Medicine, Seoul 03080, South Korea; ^18^ BIO-MAX Institute, Seoul National University, Seoul 08826, South Korea; ^19^ Interdisciplinary Program for Bioinformatics, Seoul National University, Seoul 08826, South Korea

**Keywords:** SARS-CoV-2, Syrian golden hamster, K18-hACE2 mice

## Abstract

Severe acute respiratory syndrome coronavirus 2 (SARS-CoV-2), the etiological agent of COVID-19, causes life-threatening disease. This novel coronavirus enters host cells via the respiratory tract, promoting the formation of severe pulmonary lesions and systemic disease. Few animal models can simulate the clinical signs and pathology of COVID-19 patients. Diverse preclinical studies using K18-hACE2 mice and Syrian golden hamsters, which are highly permissive to SARS-CoV-2 in the respiratory tract, are emerging; however, the systemic pathogenesis and cellular tropism of these models remain obscure. We intranasally infected K18-hACE2 mice and Syrian golden hamsters with SARS-CoV-2, and compared the clinical features, pathogenesis, cellular tropism and infiltrated immune-cell subsets. In K18-hACE2 mice, SARS-CoV-2 persistently replicated in alveolar cells and caused pulmonary and extrapulmonary disease, resulting in fatal outcomes. Conversely, in Syrian golden hamsters, transient SARS-CoV-2 infection in bronchial cells caused reversible pulmonary disease, without mortality. Our findings provide comprehensive insights into the pathogenic spectrum of COVID-19 using preclinical models.

## Teaser

**Editor's choice:** We infected K18-hACE2 mice and Syrian golden hamsters with SARS-CoV-2 and obtained comprehensive insights into COVID-19 pathogenicity in these two representative COVID-19 preclinical models.

## INTRODUCTION

Severe acute respiratory syndrome coronavirus 2 (SARS-CoV-2) emerged in 2019 and spread globally, causing the COVID-19 pandemic announced by the World Health Organization. The systemic manifestations of SARS-CoV-2 infection in humans include cough, fever, severe pulmonary diseases, neurological complications and gastrointestinal tract symptoms (Najjar et al., 2020; Sultan et al., 2020; Tian et al., 2020; Yang et al., 2020b). In recent decades, new coronaviruses have emerged with zoonotic transmission, causing life-threating disease, such as severe acute respiratory syndrome coronavirus (SARS-CoV) in 2002, Middle East respiratory syndrome coronavirus (MERS-CoV) in 2012 and SARS-CoV-2 in 2019 (Li et al., 2020; Rabaan et al., 2020). Because these virions have specific host tropism and binding receptors, a proper infection model for preclinical research is urgently needed.

These coronaviruses share similar structures, such as the nucleocapsid (N) protein, which binds to viral RNA, and the glycosylated spike (S) protein, which is expressed on the surface of virions (Rabaan et al., 2020). SARS-CoV and SARS-CoV-2 enter host cells via the binding of the S protein to the host's angiotensin-converting enzyme 2 (ACE2) receptor (Kuba et al., 2005; Bao et al., 2020), and a recent study suggested that SARS-CoV-2 primarily infects human lung ciliated cells strongly expressing ACE2 (Ahn et al., 2021). SARS-CoV-2 infection through the respiratory tract stimulates a robust immune response. Although the innate immune response is the first-line host defense system against SARS-CoV-2, the adaptive immune system, driven by B cells and T cells, is also integral to clearing the virus (Boechat et al., 2021). However, an excessive or imbalanced immune response is the driving factor leading to acute respiratory failure, viral dissemination to the distal organs and systemic PANoptosis (Giamarellos-Bourboulis et al., 2020; Hadjadj et al., 2020; Karki et al., 2021). In particular, neutrophilia is a common symptom of severe COVID-19, and excessive neutrophil production contributes to a poor prognosis and skew toward a dysregulated T-cell response (Liu et al., 2020; Parackova et al., 2021).

Suitable animal models are essential to evaluate vaccines and novel treatments against SARS-CoV-2, and to study the pathogenesis and transmission of this disease. The coronavirus is a well-known etiological agent in a large number of host species, causing a wide range of diseases, including pneumonia, villous atrophy and necrosis in the intestine, perivascular cuffing in the brain and splenocyte necrosis (Zappulli et al., 2020). SARS-CoV-2 infection in human ACE2-expressing transgenic mice (Winkler et al., 2020), ferrets (Blanco-Melo et al., 2020), Syrian golden hamsters (Chan et al., 2020) and non-human primates (Rockx et al., 2020) can mimic the pathology, immune response and clinical manifestations of COVID-19 patients. In these animal models, SARS-CoV-2 primarily infects the respiratory tract, causing mild-to-severe pulmonary disease, and may be transmitted to other organs (Blanco-Melo et al., 2020; Chan et al., 2020; Rockx et al., 2020; Winkler et al., 2020).

Among the available animal models for COVID-19, K18-promoter-driven human ACE2-expressing mice (K18-hACE2 mice) and Syrian golden hamsters have emerged as the most useful to study the pathology of SARS-CoV-2 infection, owing to their accessibility and low cost. Indeed, these two models have been applied globally for assessments of vaccines and therapeutics (Tostanoski et al., 2020; Liu et al., 2021), convalescent plasma therapy and rechallenge (Imai et al., 2020; Golden et al., 2021), and transmission dynamics (Sia et al., 2020). Both animal models are permissive to SARS-CoV-2 in the respiratory tract, as in humans, whereas some differences have been noted during infection. In previous studies, Syrian golden hamsters exhibited rapid clearance against SARS-CoV-2, and the infection was rapidly resolved (Sia et al., 2020), whereas K18-hACE2 mice showed lethality and prolonged viral replication in the respiratory tract (Oladunni et al., 2020). However, comparative data regarding the change in immune subsets, cellular tropism of SARS-CoV-2 and systemic pathogenesis during infection in these two animal models are limited, although these events appear to be important determinants of the outcomes of COVID-19 patients.

In this study, we infected K18-hACE2 mice and Syrian golden hamsters with SARS-CoV-2 and compared the clinical signs, histopathology and cellular tropism between the two models. This study provides comprehensive insights into the variations in the pathology of SARS-CoV-2 infection and could serve as a foundation for the use of preclinical models for further COVID-19-related research.

## RESULTS

### Clinical features of SARS-CoV-2-infected K18-hACE2 mice and Syrian golden hamsters

**Fig. 1.**
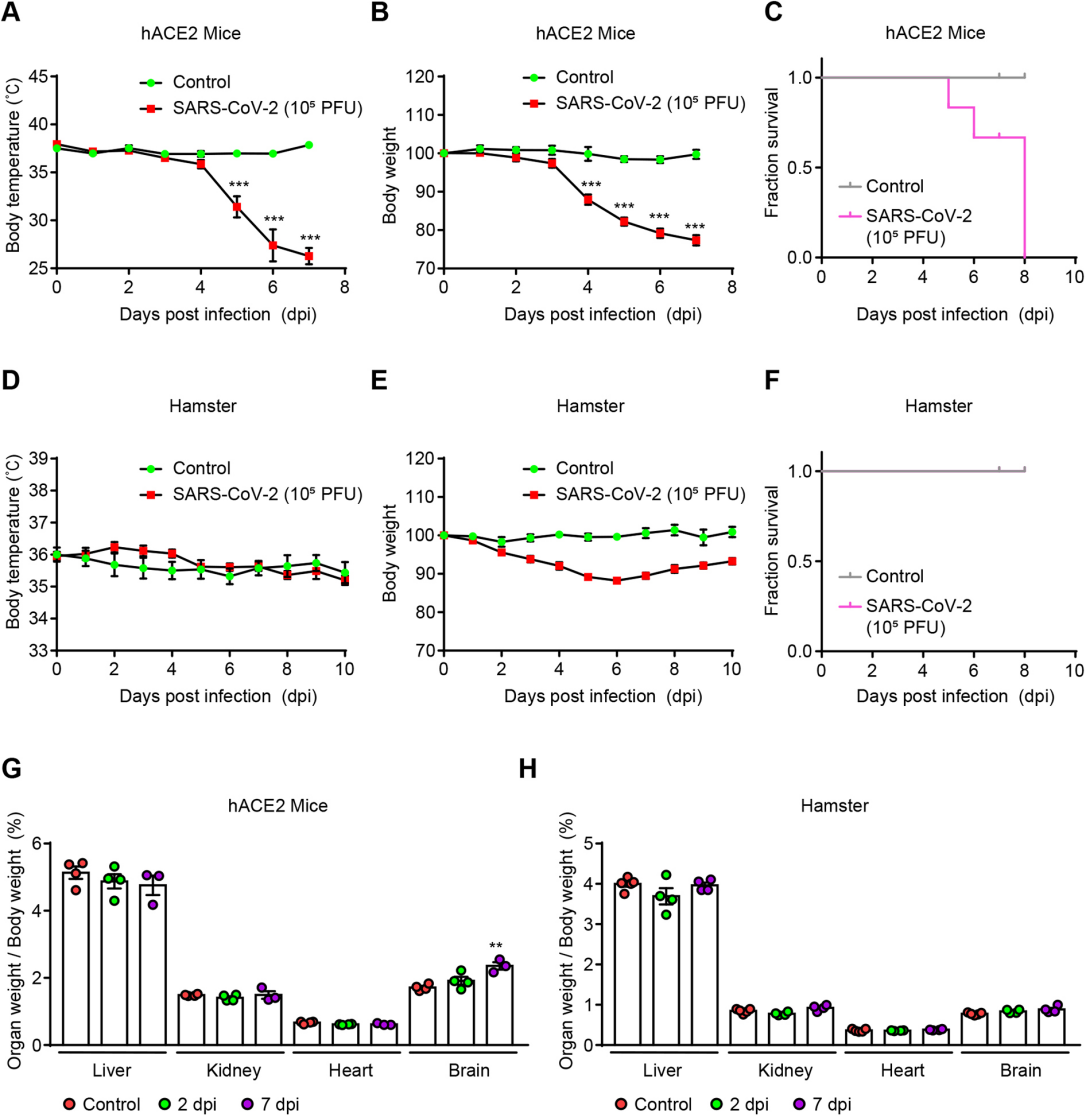
**Clinical features of SARS-CoV-2-infected K18-hACE2 mice and Syrian golden hamsters.** (A) Body temperature of SARS-CoV-2-infected K18-hACE2 mice (*n*=8) and non-infected control mice (*n*=4). *P*-values were obtained by two-tailed unpaired Student's *t*-test (***P<0.001). (B) Body weight loss for SARS-CoV-2-infected K18-hACE2 mice (*n*=8) and non-infected control mice (*n*=4). The percentage (%) represents the rate of loss compared to the initial body weight. *P*-values were obtained by two-tailed unpaired Student's *t*-test (***P<0.001). (C) Overall survival for SARS-CoV-2-infected K18-hACE2 mice (*n*=8) and non-infected controls (*n*=4). (D) Body temperature for SARS-CoV-2-infected Syrian golden hamsters (*n*=12) and non-infected controls (*n*=5). (E) Body weight loss for SARS-CoV-2-infected Syrian golden hamsters (*n*=12) and non-infected controls (*n*=5). (F) Overall survival for SARS-CoV-2-infected Syrian golden hamsters (*n*=12) and non-infected controls (*n*=5). (G) Percentage of organ weight normalized to total body weight in SARS-CoV-2-infected K18-hACE mice [*n*=3–4 per day post-infection (dpi)]. *P*-values were obtained by two-tailed unpaired Student's *t*-test (**P<0.01). (H) Percentage of organ weight normalized to total body weight in SARS-CoV-2-infected Syrian golden hamsters (*n*=4–5 per dpi). All data are presented as the mean±s.e.m. PFU, plaque-forming units.

To compare the clinical signs between K18-hACE2 mice and Syrian golden hamsters, 10^5^ plaque-forming units (PFU) of SARS-CoV-2 were intranasally administered in two models. The body temperature of the infected mice was not reduced until 4 days post-infection (dpi); however, it sharply decreased subsequently compared to that of the non-infected control group (Fig. 1A). Body weight also showed a similar reduction pattern to body temperature during SARS-CoV-2 infection (Fig. 1B), and all K18-hACE mice died within 7 dpi (Fig. 1C).

By contrast, no reduction in body temperature was observed in hamsters (Fig. 1D). Although the body weight of SARS-CoV-2-infected hamsters slightly decreased, compared with that of the control group, until 6 dpi, it had almost completely recovered by 10 dpi (Fig. 1E). In addition, none of hamsters died from SARS-CoV-2 infection throughout the experiment (Fig. 1F). There were also no significant changes in liver, kidney, heart and brain weight (relative to total body weight) in hamsters, whereas the brain weight slightly increased in SARS-CoV-2-infected K18-hACE2 mice, compared with that of the control mice, at 7 dpi (Fig. 1G,H). Collectively, the evaluation of the clinical features indicated that the severity of SARS-CoV-2 infection was worse in K18-hACE2 mice than in Syrian golden hamsters, leading to the eventual death of the mice.

### Lung pathogenesis in SARS-CoV-2-infected K18-hACE mice and Syrian golden hamsters

**Fig. 2.**
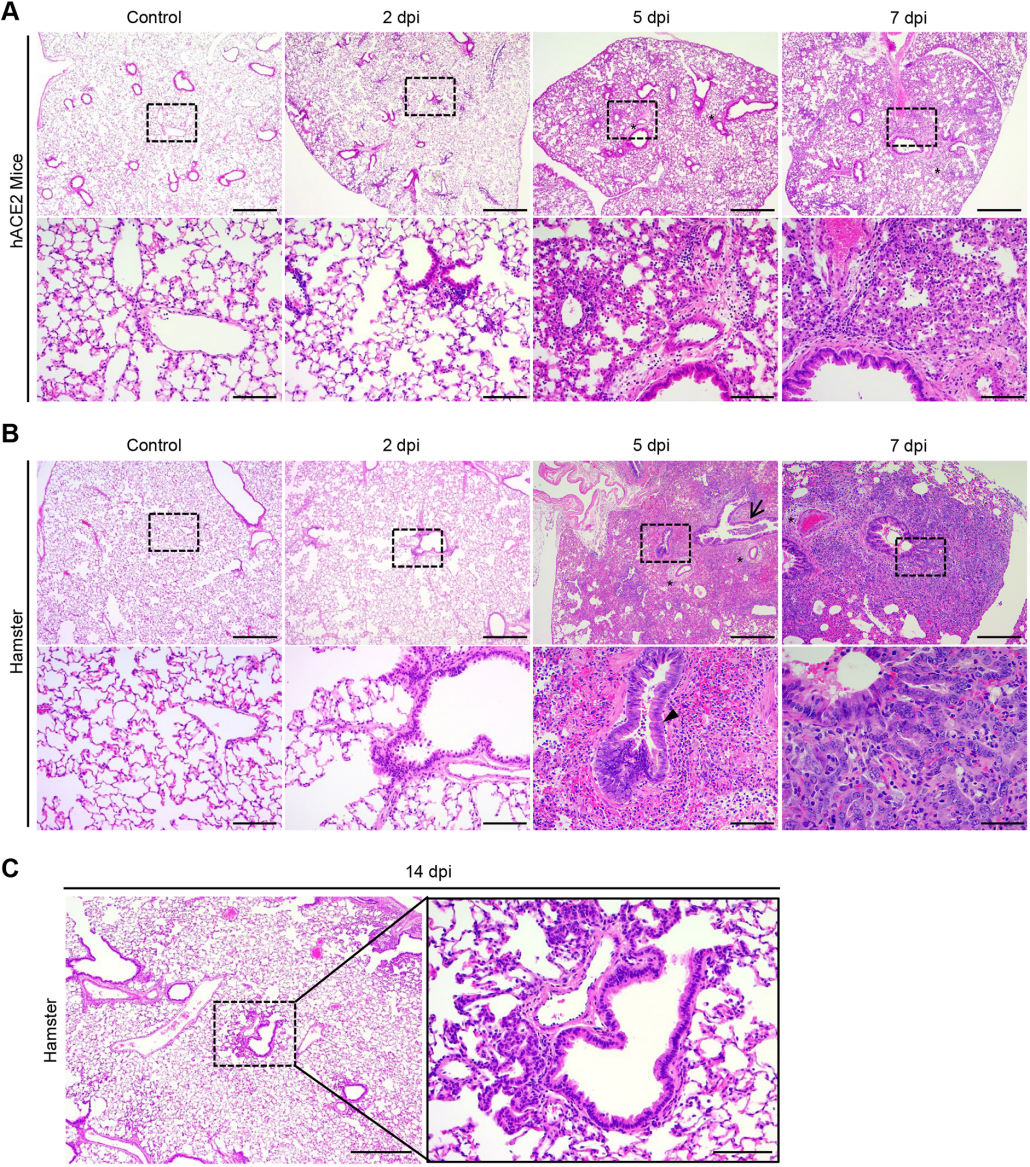
**Differential lung pathogenesis of SARS-CoV-2-infected K18-hACE2 mice and Syrian golden hamsters.** (A) Representative Hematoxylin and Eosin (H&E)-stained images of the lungs in SARS-CoV-2-infected K18-hACE2 mice and control (non-infected) mice. Scale bars: 500 µm (top row); 100 µm (bottom row). (B) Representative H&E-stained images of the lungs in SARS-CoV-2-infected Syrian hamsters and non-infected controls. Scale bars: 500 µm (top row); 100 µm (bottom row). (C) Representative H&E-stained images of the lungs in SARS-CoV-2-infected Syrian hamsters at 14 dpi. Scale bars: 500 µm (left); 100 µm (right). In the H&E images, black asterisks indicate vascular edema, the black arrowhead indicates bronchial hyperplasia, and the black arrow indicates bronchial exudates.

To further evaluate the pathogenesis of SARS-CoV-2 infection in K18-hACE2 mice and Syrian golden hamsters, the pathological observations were performed at 2, 5 and 7 dpi. Lung histopathological images showed that all K18-hACE2 mice and hamsters exhibited progressive pulmonary disease after SARS-CoV-2 infection up to 7 dpi (Fig. 2A,B).

At 2 dpi, mild lung pathology with weak inflammation was observed in the alveolar and perivascular sites in both hamsters and mice (Fig. 2A,B). Interestingly, the majority of the hamsters showed slight sloughing of the bronchial cells, exhibiting epithelial cell death in the intrabronchiole space at 2 dpi (Fig. 2B, bottom row), whereas no such signature was detected in the mice (Fig. 2A). Indeed, terminal deoxynucleotidyl transferase dUTP nick end labelling (TUNEL)-positive cells were concentrated on the bronchus in hamsters at 2 dpi (Fig. S1A). At 5 dpi, infiltration of immune cells, including mononuclear cells and neutrophils, was found at the alveolar and interstitial sites, along with vascular edema and thickening of the alveolar septa, in both K18-hACE2 mice and hamsters. Bronchial hyperplasia, along with bronchial exudates admixed with degenerative cells, was distinctly observed in hamsters, but not mice, at 5 dpi (Fig. 2B). At 7 dpi, both the mice and hamsters exhibited more progressive pathological findings than those observed at 5 dpi (Fig. 2A,B).

Overall, the severity of pulmonary disease was much more prominent in hamsters than in K18-hACE2 mice at both 5 dpi and 7 dpi (Fig. 2A,B). Bronchial epithelial cell hyperplasia and bronchopneumonia were more prominent in hamsters than in K18-hACE2 mice (Fig. 2B). In addition, accumulation of fibrin and hemorrhage were distinctly observed in hamsters at 5 dpi and 7 dpi, whereas these signatures were barely detected in mice (Fig. 2A,B). Nevertheless, all hamsters survived beyond 7 dpi, and the pulmonary disease recovered with only slight hyperplasia in the terminal bronchiole with reduced inflammatory foci at 14 dpi (Fig. 2C). Conversely, the K18-hACE2 mice died after 7 dpi despite relatively mild inflammatory cell exudates with mainly capillary dilatation in the alveolar wall of the lungs (Fig. 1C and Fig. 2A), suggesting that SARS-CoV-2 infection might elicit further damage beyond pulmonary disease features in mice. Supporting this, the numbers of TUNEL-positive cells increased remarkably in mice, with a peak at 7 dpi, whereas the increment in the number of apoptotic cells was suppressed in hamsters after showing a peak at 5 dpi (Fig. S1B,C).

**Fig. 3.**
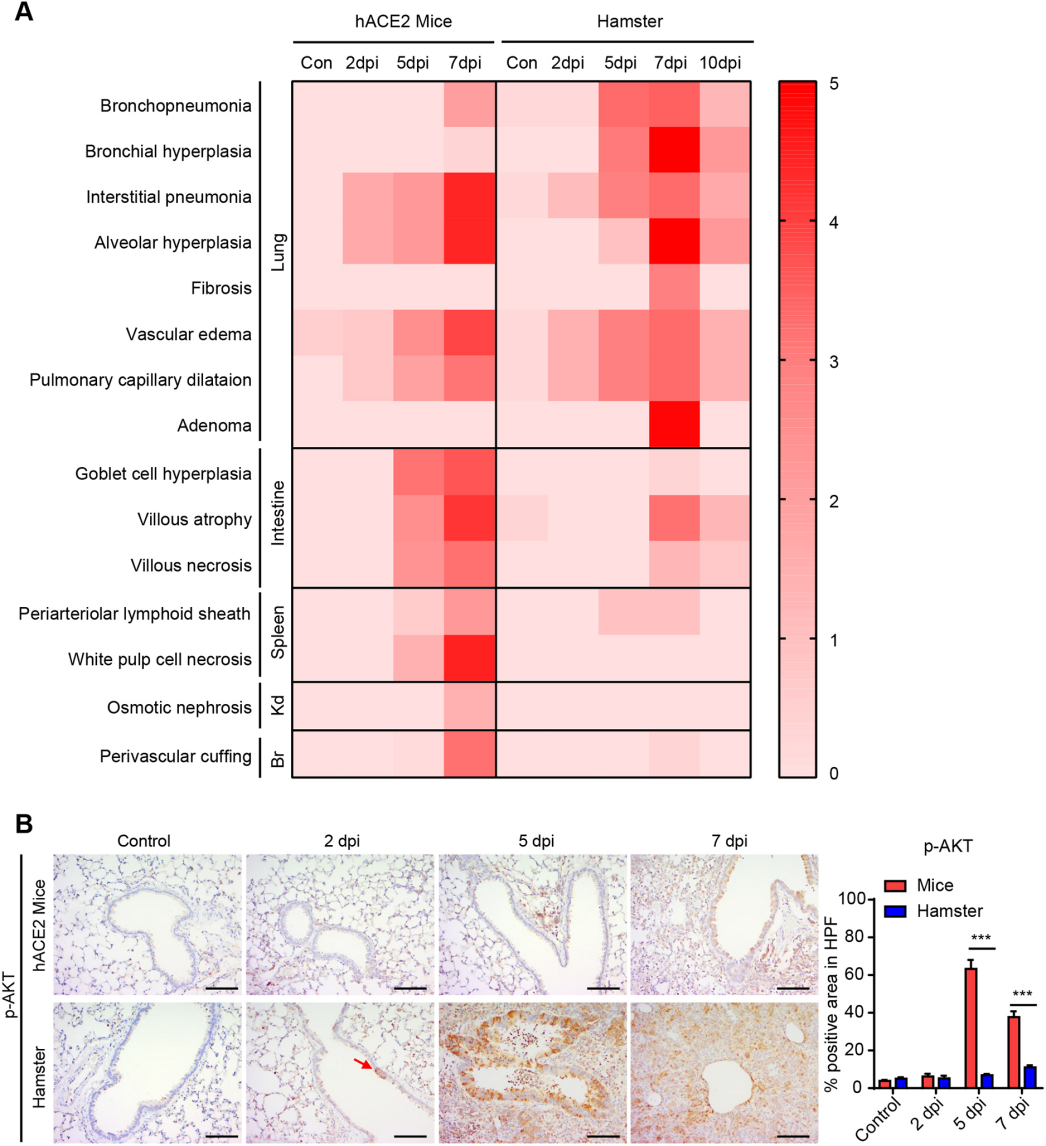
**Overall histopathological assessment of SARS-CoV-2-infected K18-hACE2 mice and Syrian golden hamsters.** (A) Heatmap showing the histopathological scores for the lung, intestine, spleen, kidney and brain in SARS-CoV-2-infected K18-hACE2 mice and Syrian golden hamsters during infection. The average score for each animal (*n*=4–5 per group) was evaluated by animal pathologists at 2, 5, 7 and 10 dpi. The severity of the pathological score ranges from 0 to 5 (0, none; 1, weak; 2, mild; 3, moderate; 4, severe; 5, markedly severe). Br, brain; Kd, kidney. (B) Representative immunohistochemical images of phosphorylated AKT (p-AKT) in SARS-CoV-2-infected K18-hACE2 mice and Syrian golden hamsters. Scale bars: 100 µm. The red arrow indicates p-AKT-positive cells in the bronchus. The graph indicates the percentage of p-AKT-positive area in a 10× high-power field (HPF) (*n*=4–5 per dpi). All data are presented as the mean±s.e.m. *P*-values were obtained by two-tailed unpaired Student's *t*-test (***P<0.001). Control represents the non-infected animals.

To evaluate the histopathological lesion severity of all animals at each time point during SARS-CoV-2 infection, we classified the pulmonary disease into eight differential terms (Fig. 3A). Bronchopneumonia and bronchial hyperplasia exclusively and progressively developed in hamsters, but no such diagnosis was made in any of the K18-hACE2 mice even at 7 dpi (Fig. 3A). Papillary bronchioloalveolar adenoma, characterized by a tubular structure with a lining of columnar cells and cellular pleomorphism, was detected in hamsters at 7 dpi (Fig. 2B and Fig. 3A). Indeed, phosphorylated AKT (p-AKT) was strongly expressed in the adenoma region, consistent with previous studies, indicating p-AKT as a lung adenoma molecular marker (Kuno et al., 2014; Hu et al., 2020) (Fig. 3B). As shown in the heatmap in Fig. 3A, the pulmonary diseases peaked at 7 dpi, with a gradual decrease in the severity score at 10 dpi in hamsters, corresponding to the histopathological images.

### Pathogenesis of the extrapulmonary organs

To find evidence of extrapulmonary disease from SARS-CoV-2 infection, all tissues were observed and diagnosed by mouse pathologists (K.T.N., J.W.P.). Specific histopathological findings were found in the spleen, kidney, intestine and brain, which were the most severe at 7 dpi (Fig. 3A; Fig. S2A–H). In the spleen, apoptotic cells characterized by nuclear pyknosis, karyolysis and karyorrhexis were clearly evident in the white pulp region of K18-hACE2 mice but not hamsters (Fig. S2A,B). The spleen lesions, including a periarteriolar lymphoid sheath and white pulp cell apoptosis, were distinct in mice, whereas these pathological features were barely observed in the hamsters (Fig. 3A; Fig. S2A,B). In the kidney, a diagnosis of focal osmotic nephrosis characterized by clear cytoplasm in the collecting duct was made in K18-hACE2 mice, but not in hamsters (Fig. 3A; Fig. S2C,D). In the small intestine, both mice and hamsters showed goblet cell hyperplasia and villous atrophy characterized by wrinkled villi and villous necrosis or loss, but the pathological score for the small intestine was higher in K18-hACE2 mice than in hamsters at 5 dpi and 7 dpi (Fig. 3A; Fig. S2E,F). Moreover, a pronounced decrease in intestinal immune cells was detected after infection in K18-hACE2 mice but not in hamsters (Fig. S3A–D). Multifocal perivascular cuffing in the brain was observed in mice, but not in hamsters, at 7 dpi (Fig. 3A; Fig. S2G,H). Taken together, these findings demonstrated that systemic histopathological damage was more pronounced in mice than in hamsters, suggesting that these changes contributed to the high mortality of K18-hACE2 mice.

### Characteristics of SARS-CoV-2 infection in K18-hACE2 mice and Syrian golden hamsters

**Fig. 4.**
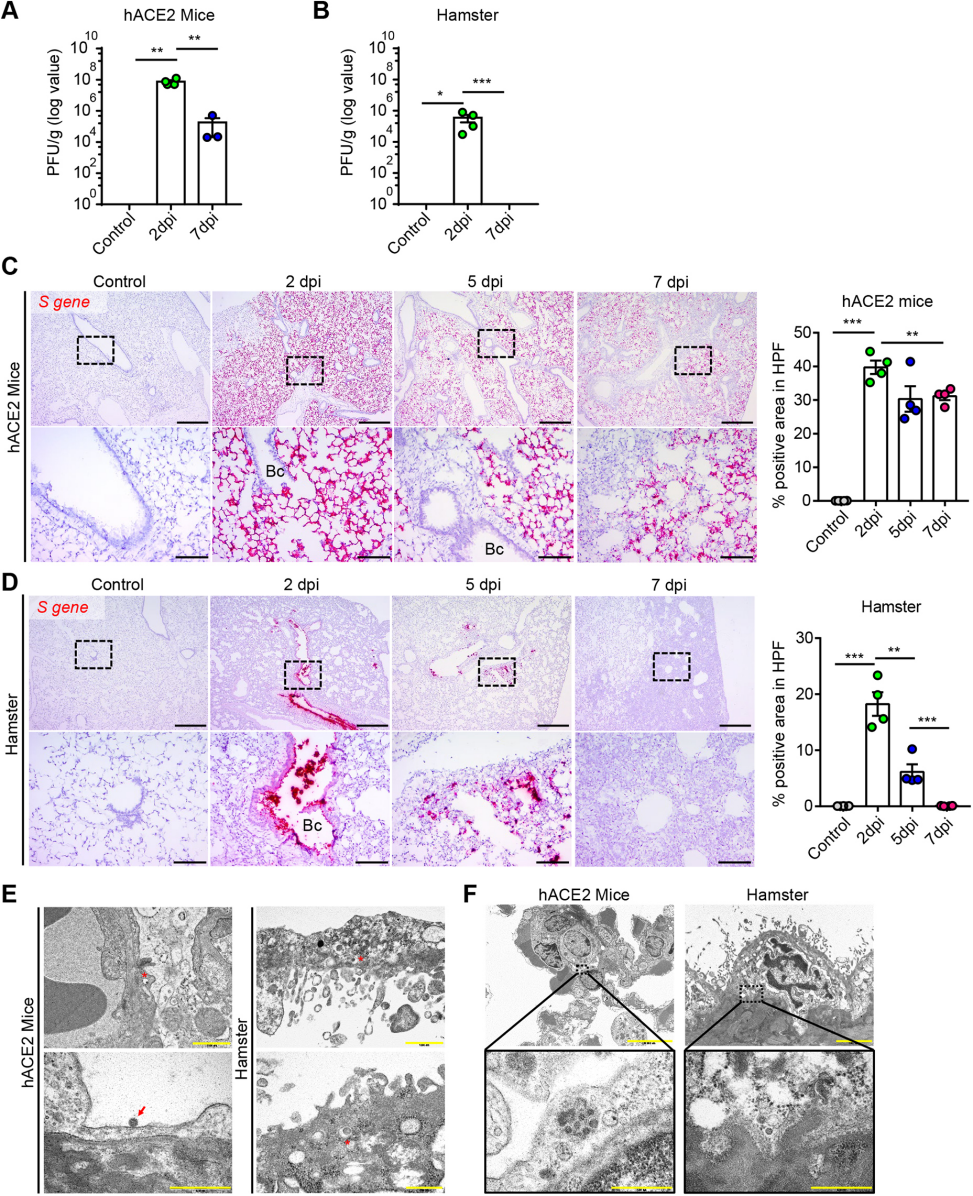
**Comparison of the characteristics of SARS-CoV-2-infected lungs.** (A,B) Viral loads in lung specimens of SARS-CoV-2-infected K18-hACE2 mice and Syrian golden hamsters (*n*=3–4 per dpi). All data are presented as the mean±s.e.m. *P*-values were obtained by two-tailed unpaired Student's *t*-test (*P<0.05; **P<0.01; ***P<0.001). (C,D) Representative *in situ* hybridization images for *S* gene in the lungs of SARS-CoV-2-infected K18-hACE2 mice and Syrian golden hamsters (*n*=4–5 per dpi). Bc, bronchus. Scale bars: 500 µm (top rows); 100 µm (bottom rows). The graph indicates the percentage *S* gene-positive area in a 10× HPF. All data are presented as the mean±s.e.m. *P*-values were obtained by two-tailed unpaired Student's *t*-test (**P<0.01; ***P<0.001). (E) Transmission electron microscopy (TEM) images of the SARS-CoV-2-infected lung in K18-hACE2 mice and Syrian golden hamsters at 2 dpi. Red asterisks indicate an encapsulated single SARS-CoV-2 virion. The red arrow indicates membrane-attached SARS-CoV-2. (F) TEM images in the alveolus of K18-hACE2 mice and bronchus of Syrian golden hamster at 2 dpi. Enlarged images show assembled SARS-CoV-2. Scale bars: 1000 nm (top left); 2000 nm (top right); 500 nm (bottom row). Control represents the non-infected animals.

To compare the infection patterns of SARS-CoV-2 in K18-hACE2 mice and Syrian golden hamsters, we performed a plaque assay and *in situ* hybridization staining using lung specimens. In K18-hACE2 mice, compared to the non-infected control group, there was a significant increase in the PFU value at 2 dpi and 7 dpi, and the virus remained in the late stage of infection (Fig. 4A). By contrast, in the hamsters, there was a negative PFU value of SARS-CoV-2 at 7 dpi despite a significant increase at the early stage of infection (Fig. 4B). The *in situ* hybridization images indicated that SARS-CoV-2-infected cells were distributed throughout the alveolar region of the lung in mice, even at 2 dpi, and still existed in identical loci at 5 dpi and 7 dpi (Fig. 4C), supporting the high PFU values at the late stage of infection. However, SARS-CoV-2-infected cells were barely detected in the bronchus and inflammatory cells of mice during the infection (Fig. 4C). In hamsters, SARS-CoV-2-infected cells were concentrated on the bronchus and in the intrabronchiole region at 2 dpi and 5 dpi, and were not detected distal to the bronchus (Fig. 4D). We also observed clear difference in the infection area between mice and hamsters infected with high-dose (1×10^6^ PFU) SARS-CoV-2 (Fig. S4A). Surprisingly, in the hamsters, SARS-CoV-2-infected cells disappeared from the entire pulmonary region despite the existence of severe pulmonary inflammation and pathological lesions (Fig. 4D).

Ultrastructure images of SARS-CoV-2-infected lungs clearly demonstrated that a single virion was attached to the cell membrane or entered the host cell by endocytosis in K18-hACE2 mice and hamsters, respectively, at 2 dpi (Fig. 4E). In addition, the assembled virions were detected in the pneumocytes of mice and in the microvilli-containing bronchial epithelial cells of hamsters (Fig. 4F). Interestingly, the cells infected with SARS-CoV-2 were detached from the bronchial wall and appeared degenerated in hamsters (Fig. 4E,F). These results substantiated that the distribution of the primary infection site was clearly different between the two animal models, and SARS-CoV-2 gradually disappeared in hamsters, unlike in K18-hACE2 mice.

As expected, SARS-CoV-2-infected cells were not detected in organs other than the pulmonary organs in the hamsters, at both the early and late stages of infection (Fig. S5B). Only a few infected cells were found in the spleen at 2 dpi (Fig. S5B). Likewise, there were no SARS-CoV-2-infected cells in the kidney, intestine and liver of the mice at 2 dpi and 7 dpi (Fig. S5A). However, a substantially high number of *S* gene-positive splenocytes were present in the white pulp at 2 dpi in mice, and the number decreased at 7 dpi (Fig. S5A). Although there was no SARS-CoV-2 detected in the brain at 2 dpi, strong expression of the *S* gene was focally observed in the neuronal cells at 7 dpi (Fig. S5A), indicating the dissemination of SARS-CoV-2 in the late stage of infection in K18-hACE2 mice.

### Identification of SARS-CoV-2-infected cells in K18-hACE2 mice and Syrian golden hamsters

We postulated that the clear differences in the primary infection site between K18-hACE2 mice and Syrian golden hamsters (Fig. 4C,D) may contribute to the differences in the severity of the histopathological phenotype between the two models. To understand the cells that were preferentially infected with SARS-CoV-2 in the two animal models, we performed dual immunostaining for N protein and representative lung lineage markers. We first confirmed no distinct change between RNA (*S* gene) and protein (N protein) expression for SARS-CoV-2 in the brain and the lung (Fig. S6A).

**Fig. 5.**
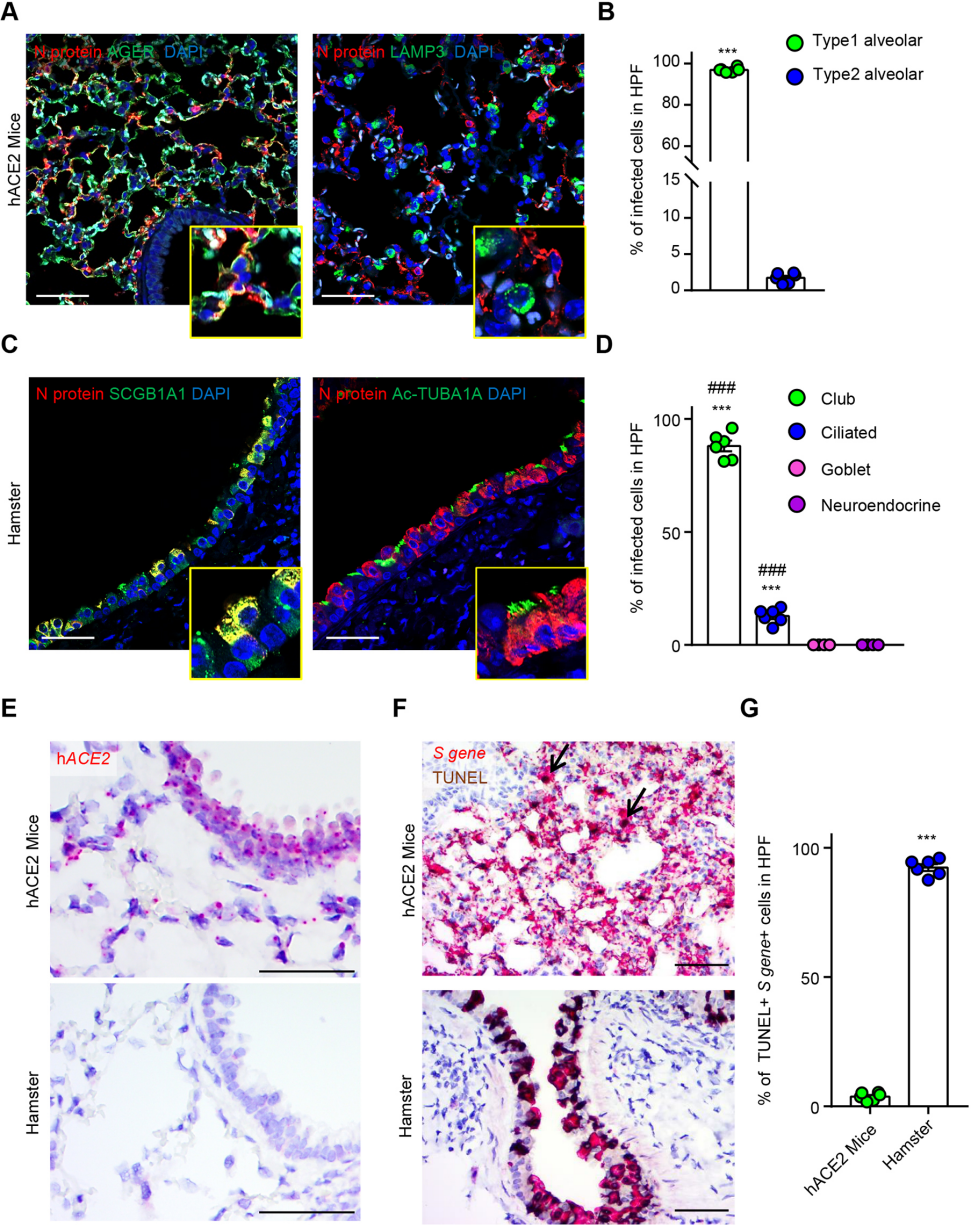
**Identification of SARS-CoV-2-infected cells in the lungs of K18-hACE2 mice and Syrian golden hamsters.** (A) Representative immunofluorescence images for N protein, AGER (type 1 alveolar cell marker), LAMP3 (type 2 alveolar cell marker) and 4′,6-diamidino-2-phenylindole (DAPI; nuclear marker) in the lungs of K18-hACE2 mice 2 days after SARS-CoV-2 infection. Scale bars: 40 µm. (B) Percentage of infected cells (N protein^+^) per total positive cells in the alveolar lineage (AGER^+^ or LAMP3^+^) in SARS-CoV-2-infected K18-hACE2 mice at a 40× HPF (*n*=6 per group). All data are presented as the mean±s.e.m. *P*-values were obtained by two-tailed unpaired Student's *t*-test (***P<0.001). (C) Representative immunofluorescence images for N protein, SCGB1A1 (club cell marker), acetylated TUBA1A (Ac-TUBA1A; ciliated cell marker) and DAPI in the lungs of Syrian golden hamsters 2 days after SARS-CoV-2 infection. Scale bars: 40 µm. (D) Percentage of infected cells (N protein^+^) per total number of positive cells in the bronchus lineage (SCGB1A1^+^ or Ac-TUBA1A^+^ or MUC5AC^+^ or CHGA^+^) in SARS-CoV-2-infected Syrian golden hamsters in a 40× HPF (*n*=6 per group). All data are presented as the mean±s.e.m. *P*-values were obtained by two-tailed unpaired Student's *t*-test (***P<0.001, versus goblet cells; ^###^*P*<0.001, versus neuroendocrine cells). (E) Representative *in situ* hybridization images of human *ACE2* (h*ACE2*) in the lungs of non-infected K18-hACE2 mice and Syrian golden hamsters. Scale bars: 50 µm. (F) Co-staining images of *in situ* hybridization for *S* gene and immunohistochemical images of TUNEL in the lungs of K18-hACE2 mice and Syrian golden hamsters 2 days after SARS-CoV-2 infection. The black arrows indicate TUNEL^+^/*S* gene^+^ cells. Scale bars: 50 µm. (G) Percentage of TUNEL^+^/*S* gene^+^ cells relative to total *S* gene^+^ cells in SARS-CoV-2-infected K18-hACE2 mice and Syrian golden hamsters in a 40× HPF (*n*=6 per group). All data are presented as the mean±s.e.m. *P*-values were obtained by two-tailed unpaired Student's *t*-test (***P<0.001).

In K18-hACE2 mice, the N protein-positive cells were enriched in the alveolar region and preferentially infected type 1 alveolar cells (Fig. 5A,B). Approximately 90% of the total N protein-positive cells were type 1 alveolar cells (Fig. 5B). However, less than 2% of the type 2 alveolar cells comprised SARS-CoV-2-infected cells. In hamsters, the majority of the subset of SARS-CoV-2-infected cells were club cells, with a few infected ciliated cells (Fig. 5C,D). The goblet and neuroendocrine cells were not infected with SARS-CoV-2 (Fig. 5D; Fig. S6B). Surprisingly, human *ACE2* expression was detected not only in the alveolar cells but also in the bronchial cells in K18-hACE2 mice, and *ACE2* expression was absent in hamsters as expected (Fig. 5E).

Consistently, the majority of the SARS-CoV-2-infected cells had died in hamsters at 2 dpi, whereas the rate of cell death was much lower in mice at the same time point (Fig. 5F,G). Overall, there were substantial differences with regard to the virus-infected cell subsets and consequences to the infected cells in the two models.

### Comparison of immune subsets of SARS-CoV-2-infected lungs

**Fig. 6.**
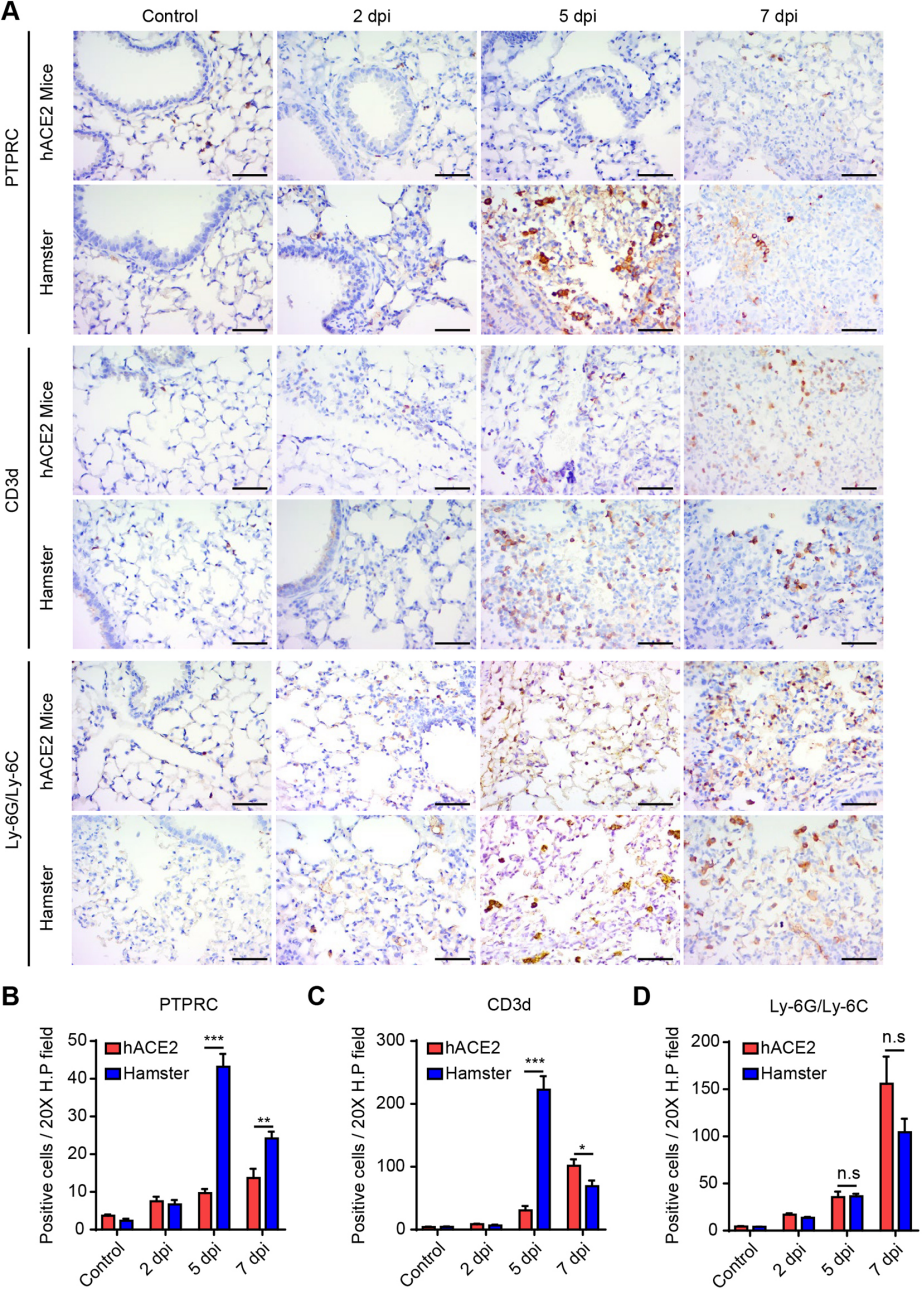
**Comparison of pulmonary immune cell subsets in SARS-CoV-2-infected K18-hACE2 mice and Syrian golden hamsters.** (A) Representative immunohistochemical images of PTPRC (B-cell marker), CD3D (T-cell marker) and Ly-6G/Ly-6C (neutrophil marker) in SARS-CoV-2-infected K18-hACE2 mice and Syrian golden hamsters. Scale bars: 40 µm. (B–D) Numbers of PTPRC-positive (B), CD3D-positive (C) and Ly-6G/Ly-6C-positive (D) cells in a 20× high-power (H.P.) field (*n*=6 per group). Control represents the non-infected animals. *P*-values were obtained by two-tailed unpaired Student's *t*-test (*P<0.05; **P<0.01; ***P<0.001; n.s., not significant). All data are presented as the mean±s.e.m.

Following SARS-CoV-2 infection, there is an immense inflammatory response throughout the pulmonary region (Li et al., 2020; Winkler et al., 2020). Therefore, we compared representative immune subsets after SARS-CoV-2 infection in the two animal models. The numbers of PTPRC-positive B cells and CD3D-positive T cells increased significantly in hamsters at 5 dpi, compared with those in K18-hACE2 mice at the same time point (Fig. 6A–C). In hamsters, the number of adaptive immune cells increased in the lung to the greatest extent at 5 dpi, and sharply declined at 7 dpi, when the virus was eliminated throughout the pulmonary region (Fig. 6A–C). In K18-hACE2 mice, the number of B cells in the lung progressively increased, although the degree of infiltration was lower than that in hamster (Fig. 6A,B). Similar to the change in B-cell numbers, the numbers of T cells also progressively increased during SARS-CoV-2 infection in K18-hACE2 mice (Fig. 6A,C). The numbers of Ly-6G/Ly-6C (also known as Ly-6C2)-positive neutrophils progressively increased in both models during SARS-CoV-2 infection to the same degree (Fig. 6A,D). Because the CD4-to-CD8 ratio and neutrophil-to-CD8 ratio have been identified as reliable risk factors for predicting COVID-19 mortality (Pallotto et al., 2020; De Zuani et al., 2022), we further analyzed T-cell subsets (Fig. S7A–C). The results showed a prominent increase in CD8-positive cells in hamsters at 5 dpi, and the CD4-to-CD8 ratio and neutrophil-to-CD8 ratio were 1.8-fold and 3.2-fold higher, respectively, in K18-hACE2 mice than in hamsters at the same time point.

## DISCUSSION

As human clinical research for SARS-CoV-2 is complicated owing to variations due to genetic diversity, host age and the wide range of disease severity, including asymptomatic patients, preclinical models are essential to study the pathogenesis and to develop therapeutics and antiviral vaccines (Pandey et al., 2020). Small-animal models, including K18-hACE2 mice and Syrian golden hamsters, have been globally utilized to assess vaccines, as well as the pathogenesis and transmissibility of SARS-CoV-2 (Pandey et al., 2020; Tostanoski et al., 2020; Winkler et al., 2020; Liu et al., 2021). These models develop infections in both the upper and lower respiratory tracts, as is the case in humans, and also mimic the severe pulmonary disease and inflammatory response seen in COVID-19 patients (Brocato et al., 2020; Chan et al., 2020; Oladunni et al., 2020; Winkler et al., 2020). In the present study, our comparative analysis elucidated the marked differences between these two models with regard to clinical features, pathogenesis, cellular tropism and immune-cell subsets against SARS-CoV-2.

Reliable research on COVID-19 patients has shed light on the cellular tropism of SARS-CoV-2 in the respiratory tract. Ciliated cells, pulmonary pneumocytes and club cells appear to be the major targets of SARS-CoV-2 in the respiratory tract of humans (Hui et al., 2020; Schaefer et al., 2020; Ahn et al., 2021). However, the cellular tropism of SARS-CoV-2 in animal models has yet to be defined. We found that specific cells, especially those in the lung parenchyma, were preferentially and primarily infected with SARS-CoV-2 in both mice and hamsters. However, in the hamsters, *in situ* hybridization images demonstrated that SARS-CoV-2 infection was confined to the bronchus region despite a few *S* gene-positive cells being detected in the peribronchial alveolar cells at 5 dpi. Of note, the major subset of SARS-CoV-2-infected cells was the microvillar club cell subset (∼88%), followed by the ciliated cell subset (∼12%). This observation was consistent with the ultrastructure images, although no quantitative results could be obtained for direct comparison. Mucus-secreting goblet cells and neuroendocrine cells were not infected with SARS-CoV-2 in the hamsters. By contrast, in the K18-hACE2 mice, the SARS-CoV-2-infected region in the lung was mainly confined to the alveolar area rather than to the bronchus region. Indeed, SARS-CoV-2-infected cells in the K18-hACE2 mice exclusively comprised type 1 alveolar cells (∼97%), representing a vastly different distribution pattern from that of the hamsters.

SARS-CoV-2 enters the host cell by binding to the host's ACE2 receptor (Kuba et al., 2005; Bao et al., 2020). The *in situ* hybridization images indicated that *ACE2* was strongly expressed in the alveolar cells as well as in the bronchus cells that were not infected with SARS-CoV-2 in K18-hACE2 mice. It is noteworthy that SARS-CoV-2 did not have cellular tropism toward bronchus-lineage cells despite the high *ACE2* expression in K18-hACE2 mice. Although hamsters did not express *ACE2* throughout the lung parenchyma, previous *in silico* studies substantiated that hamster ACE2 could bind to the S protein of SARS-CoV-2, thereby facilitating host cell infection (Chan et al., 2020; Luan et al., 2020).

Consistent with previous studies (Chan et al., 2020; Imai et al., 2020; Sia et al., 2020), early eradication of SARS-CoV-2-infected cells was observed in hamsters. To investigate the fate of SARS-CoV-2-infected cells, we performed double staining for TUNEL and *S* gene in both animal models. Surprisingly, in contrast to observations in K18-hACE2 mice, the *S* gene-positive bronchus cells were quickly eliminated at 2 dpi in hamsters. In addition, immune cells rapidly accumulated during early infection; this accumulation was ameliorated after viral clearance. This finding implies that the early clearance of the virus in hamsters is prompted by immune cell-mediated cytotoxicity, which subsequently hampers the ongoing replication of the virus in the respiratory tract. Moreover, *S* gene-positive alveolar cells were rarely eliminated in K18-hACE2 mice despite immune cell infiltration, suggesting that bronchus cells may be more susceptible to cytotoxicity than alveolar cells. Zhou et al. (2021) revealed that patients with late viral clearance had more severe and systemic lesions compared to those of patients with early viral clearance. Consistently, SARS-CoV-2 was sustained in the lung parenchyma of K18-hACE2 mice until death (7 dpi) and eventually disseminated to the distal organs. The extrapulmonary disease/pathology was remarkably more severe in the K18-hACE2 mice than in hamsters. In particular, a strong SARS-CoV-2 signal was detectable at 7 dpi in the brains of K18-hACE2 mice, but not in the brains of hamsters. A previous study showed that specific delivery of adeno-associated virus-hACE2 to the brain resulted in weight loss and lethality after SARS-CoV-2 infection (Song et al., 2021), and another study revealed that transmission of SARS-CoV-2 to the brain was only observed in the deceased mice (Jiang et al., 2020). These previous results support our speculation that the difference in clinical signs between K18-hACE mice and hamsters may be linked to the neuroinvasion of SARS-CoV-2 in the two models.

T cells play critical roles in eliminating viral-infected cells as well as in promoting immunopathogenesis (Li et al., 2020). CD8^+^ T cells directly kill infected cells, and CD4^+^ T cells enable CD8^+^ T-cell and B-cell activation. Patients with SARS-CoV-2-specific T cells tend to show better clinical outcomes (Thevarajan et al., 2020). Consistent with this finding, animal models lacking B cells and T cells showed aggravated pulmonary disease and regressed coronavirus clearance compared to immunocompetent controls (Zhao et al., 2014; Brocato et al., 2020). Indeed, during SARS-CoV-2 infection, the degree of increase or decrease in lung apoptosis was very similar to that of T cells in both K18-hACE2 mice and hamsters. Therefore, it can be predicted that a rapid increase in T-cell numbers enables the early eradication of SARS-CoV-2 in Syrian golden hamsters, contributing to a non-fatal disease course. Neutrophils are innate immune cells responding to viral infection; neutrophilia (excessive neutrophil production) is associated with the severity of SARS-CoV-2 (Wang et al., 2020). In addition, an elevated neutrophil-to-lymphocyte ratio (NLR) in the peripheral blood is consistently found to be an independent risk factor for COVID-19 severity (Henry et al., 2020; Liu et al., 2020; Yang et al., 2020a), which has also been found in the lung airway (Radermecker et al., 2020). The NLR of the lung airway was higher in K18-hACE2 mice than in hamsters at 5 dpi (0.88 versus 0.13) and 7 dpi (1.36 versus 1.12) (Fig. 6B–D). A higher NLR was also observed in the blood of K18-hACE2 mice than in the blood of hamsters at 7 dpi (0.96 versus 0.76) (Table S1). Hence, this unbalanced immune subset is also likely responsible for the poor outcomes in K18-hACE2 mice. However, further experiments are needed to test this hypothesis, including T-cell sorting or use of a genetically engineered animal model.

Despite the remarkably poor clinical outcomes in mice, the pulmonary disease/pathology was more severe in hamsters at 5 dpi and 7 dpi. At those time points, B cells and T cells accumulated to a greater degree in Syrian golden hamsters than in K18-hACE2 mice, suggesting that severe disease was accelerated by these immune cells in hamsters given that T cells induce immunopathology (Brocato et al., 2020; Li et al., 2020). Of note, we observed an area showing positive staining for p-AKT – which is strongly expressed during lung carcinogenesis (Hu et al., 2020) – that extended from the bronchus prone to SARS-CoV-2 infection. Despite this reversible phenotype, we diagnosed the area as a pulmonary adenoma, given the proliferating tubular structures with apparent pleomorphic cells. However, after the infiltration of adaptive immune cells was alleviated, the formation of pulmonary lesions, including the adenoma, was rapidly ameliorated; only weak bronchus hyperplasia, which is a regenerative process secondary to injury, was observed at 14 dpi (Suttie et al., 2018). These results suggest that radical immune cell infiltration contributes to viral clearance, and also to a temporarily severe pulmonary pathology in hamsters. By contrast, in K18-hACE2 mice, the accumulation of immune cells was not alleviated, in parallel with the prolonged replication of the virus throughout the lung parenchyma until death (7 dpi). A recent study revealed that an excessive immune reaction in K18-hACE2 mice induced PANoptosis (Karki et al., 2021), and this signature was recapitulated in the intestine and spleen in our study (Fig. 3). This finding indicates that the sustained infiltration of immune cells due to the late clearance of the virus led to a dysregulated immune response in K18-hACE2 mice, causing mortality and cell death.

## MATERIALS AND METHODS

### Animals

K18-hACE2 mice from a C57BL/6 background (strain #034860) were purchased from The Jackson Laboratory, and Syrian golden hamsters were purchased from Janvier Laboratories. All animal experiments were conducted in accordance with the Public Health Service and Humane Care and Use of Laboratory Animal and Association for Assessment and Accreditation of Laboratory Animal Care (AALAC)-accredited unit (#001071). The experimental protocols were approved by the Institutional Animal Care and Use Committees (IACUC; 2020-0216, BA-2008-301-071-03) at Yonsei University College of Medicine and Seoul National University Bundang Hospital. All procedures involving animals, including SARS-CoV-2 infection, were conducted in a Biosafety Level 3 facility in accordance with safety guidelines.

To infect SARS-CoV-2 in the animal models, 9-week-old male K18-hACE2 mice and 13-week-old male Syrian golden hamsters were anesthetized with a zoletil-rompun mixture (4:1), and then intranasally administered 50 µl virus-containing medium (1×10^5^ PFU). Their body temperature and body weight were measured daily after SARS-CoV-2 infection until the animals died. Body temperature was measured using an implantable programmable temperature transponder (IP55-300, BMDS). For total blood cell counts, the blood was collected with a 1-ml syringe and then 50–100 µl of blood was placed in 1.5-ml microtubes containing 20 µl of 0.5 M ethylenediaminetetraacetic acid to avoid blood clotting. The complete blood count was obtained using a hematology analyzer (BC-5000, Mindray Global).

### Virus and cells

Vero cells, cells from an African green monkey kidney cell line (KCLB 10081), were purchased from the Korean Cell Line Bank. Vero cells were cultured in Dulbecco's minimum essential medium (DMEM) supplemented with 2 mM l-glutamine, 100 units/ml penicillin, 100 µg/ml streptomycin and 5% fetal bovine serum (FBS) at 37°C in a 5% CO_2_ humidified incubator. SARS-CoV-2 (NCCP 43326, Wuhan strain) was purchased from the National Culture Collection for pathogens of Osong, South Korea. The virus was cultured in 75-cm^2^ culture flasks in Vero cells and titrated by a plaque assay on six-well plates with monolayered Vero cells according to a standard protocol. For virus growth, the cells were seeded in 75-cm^2^ culture flasks at a density of 3×10^5^ cells/ml. After 18–20 h, the subconfluent cell monolayer was washed with sterile Dulbecco's phosphate-buffered saline (PBS). The cells were infected with 2 ml serum-free DMEM containing the virus at a multiplicity of infection of 0.001 and 0.01. After 1 h of incubation at 37°C in a humidified atmosphere with 5% CO_2_, 12 ml DMEM containing 2% FBS was added to the Vero cells. The flasks were observed daily, and the virus was harvested when over 80% of the cells manifested a cytopathic effect. The culture medium was centrifuged at 4°C and 380 ***g*** for 15 min to remove the cell debris, aliquoted and stored at −80°C.

For the plaque assay, Vero cells were seeded in a six-well plate the day prior to the assay and infected with virus samples serially diluted in a serum-free medium for 1 h with gentle agitation every 15 min. The cells were then overlaid with DMEM containing 1% SeaPlaque™ agarose (Lonza), 2% FBS, 100 U/ml penicillin and 100 µg/ml streptomycin. After incubation for 3 days, when clear plaques were observed, the cells were fixed with 4% paraformaldehyde and stained with a 0.5% Crystal Violet/20% methanol solution. The plaques were counted and multiplied by the dilution factor to determine the virus titer.

### Histopathological analysis

All tissues, including the lungs, were collected for pathological observations. For Hematoxylin and Eosin staining, the slides were dipped in 0.1% Mayer's Hematoxylin for 10 min and then in 0.5% Eosin. After staining, the following sequential washing steps were performed: distilled water until the Eosin stopped streaking, dip in 50% ethanol ten times, dip in 70% ethanol ten times, 95% ethanol for 30 s and 100% ethanol for 1 min. The sections were covered with mount solution (Thermo Fisher Scientific), and the pathological scores and diagnoses were determined by experienced mouse pathologists (K.T.N. and J.W.P.).

The heatmap of pathological data was constructed using GraphPad Prism software in which each spot represents the average pathological score for K18-hACE2 mice and Syrian golden hamsters.

### *In situ* hybridization

For *in situ* hybridization staining, RNA probes (human *ACE2* and SARS-CoV-2 *S* gene) and the required reagents were commercially purchased from RNAscope from ACD (Newark, CA, USA), and *in situ* hybridization was performed according to the manufacturer's protocol. In brief, the paraffin sections were deparaffinized in xylene and the slides were incubated in 100% ethanol two times. After air drying, each step, including hydrogen peroxide treatment, incubation in target retrieval solution and protease K treatment, was performed precisely using the reagents from the kit (ACD). The RNA signal was amplified using amplifying reagent (ACD) after 2 h of probe incubation and was detected with Fast Red reagent (ACD).

### Immunohistochemistry

Paraffin sections with a 4-µm width were deparaffinized in xylene three times, rehydrated in a descending grade of ethanol (100% three times, 95% two times, 70% one time) and immersed in distilled water. The antigen retrieval step was then performed using pH 6.0 citrate buffer (DAKO) for 15 min under a high-pressure cooker, and the sections were immediately cooled in an ice bucket for at least 1 h after antigen retrieval. The sections were incubated in 3% H_2_O_2_ for 30 min to block endogenous peroxidase, washed twice with PBS and incubated with blocking buffer (DAKO) for 2 h at room temperature. The sections were treated with M.O.M. (Vector Laboratories) reagent for 1 h when the host of the primary antibody was mouse. The sections were incubated with the following primary antibodies overnight at 4°C in a humidity chamber: anti-N protein (NB100-56576, Novus, 1:1000 and 40143-MM08, Sino Biological, 1:1000), anti-LAMP3 (DDX0190P, Novus, 1:500), anti-AGER (MAB1179, R&D Systems, 1:200), anti-SCGB1A1 (ABS1673, Millipore, 1:200), anti-acetylated TUBA1A (T7451, Sigma-Aldrich, 1:1000), anti-MUC5AC (MA5-12178, Thermo Fisher Scientific, 1:1000), anti-PTPRC (ab64100, Abcam, 1:1000), anti-CD3D (ab5690, Abcam, 1:200), anti-Ly-6G/Ly-6C (ab2557, Abcam, 1:250), anti-p-AKT (9271S, Cell Signaling Technology, 1:500), anti-CHGA (ab15160, Abcam, 1:1000), anti-CD4 (25229S, Cell Signaling Technology, 1:200) and anti-CD8 (14-0808-82, Thermo Fisher Scientific, 1:200). The sections were then incubated in horseradish peroxidase (HRP)-conjugated secondary antibody (DAKO) for 15 min at room temperature. The immunohistochemical signal was developed using DAB substrate (DAKO), and Mayer's Hematoxylin was used for nuclear staining. For immunofluorescence staining, primary antibodies were detected with Alexa Fluor 488-conjugated anti-mouse, rat and goat IgG (Invitrogen), and Cy3-conjugated anti-rabbit IgG (Invitrogen). The immunofluorescence images were acquired using EVOS-FL (Invitrogen) and LSM 980 (Zeiss) systems.

### Transmission electron microscopy

Lung specimens with a 1-mm^2^ area were fixed for 24 h in 2% glutaraldehyde/paraformaldehyde in 0.1 M PBS and washed in 0.1 M phosphate buffer. The skins were post-fixed with 1% OsO_4_ dissolved in 0.1 M phosphate buffer for 2 h, dehydrated in an ascending grade of ethanol and infiltrated with propylene oxide. The skins were then embedded using a Poly/Bed 812 kit (Polysciences). After pure fresh resin embedding and polymerization at 65°C in a vacuum oven for 24 h, sections of 200–250 nm thickness were initially cut and stained with Toluidine Blue (Sigma-Aldrich) for light microscopy. Sections (70-nm-thick) were double stained with 6% uranyl acetate for 20 min and with lead citrate for contrast staining. The sections were cut using a Leica EM UC-7 system with a diamond knife and transferred onto copper and nickel grids. All of the sections were examined under a transmission electron microscope (JEOL) at 80 kV.

### TUNEL staining

To detect immunofluorescence staining for TUNEL, a Click-iT^®^ Plus TUNEL Assay kit (Thermo Fisher Scientific) was used according to the manufacturer's protocol. EdUTP was conjugated by TdT enzyme in the lung specimens and detected with Alexa Fluor 488. Immunofluorescence images were taken by the EVOS-FL system.

For co-staining of TUNEL and *S* gene, *in situ* hybridization was performed preferentially prior to the TUNEL staining according to the RNAscope^®^ manual as described above, and the TUNEL signal was developed using a TACS-XL *in situ* apoptosis detection kit (R&D Systems). In brief, after detection of the RNA signal by Fast Red, the slides were immediately washed with PBS three times and were immersed in quenching solution. B-dNTP was then conjugated by incubation of TdT enzyme and Strep-HRP solution at room temperature. The TUNEL signal was developed by immersing the slides in DAB solution.

### Statistical analysis

Statistical analysis was performed using GraphPad Prism software v7.0 with at least three samples analyzed per group. Statistical significance was obtained using unpaired Student's *t-*test (two-tailed) or one-way analysis of variance with Dunnett's multiple comparison. All data and graphs are presented as the mean±s.e.m.; differences with *P<*0.05 were considered statistically significant.

## Supplementary Material

10.1242/dmm.049632_sup1 Supplementary information
