# Supplementary material for: Comparison of the pathogenesis of SARS-CoV-2 infection in K18-hACE2 mouse and Syrian golden hamster models
Source: Dis Model Mech. 2022 Nov 11;15(11):dmm049632. doi: 10.1242/dmm.049632 (PMC9672931; doi:10.1242/dmm.049632)
Supplement: Supplementary information [file dmm-15-049632-s1.pdf]

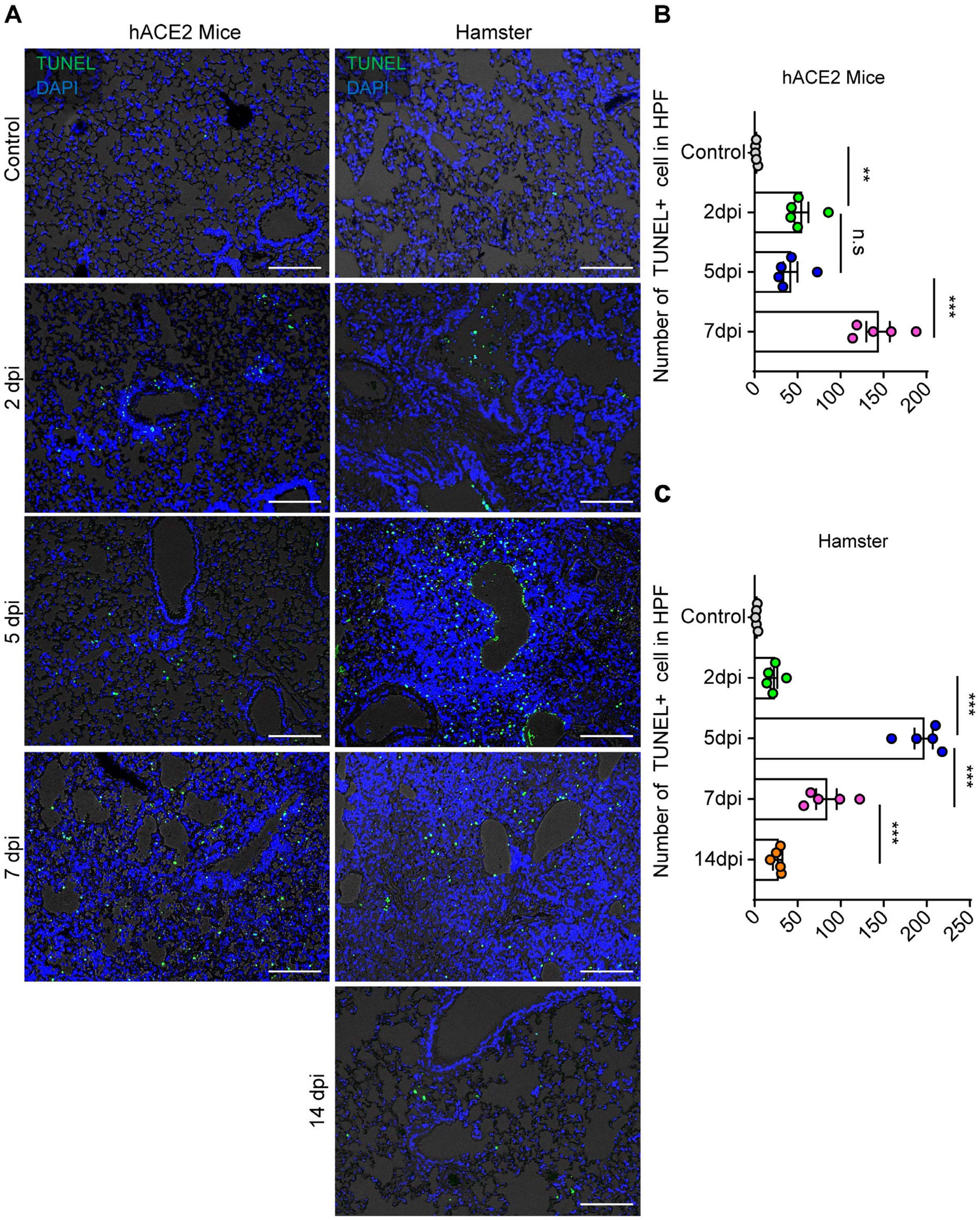

Fig. S1. Dynamic comparison of apoptotic cells in the SARS- CoV-2-infected lungs. (A)

Representative immunofluorescence images for TUNEL and DAPI staining in K18-hACE2

Disease Models & Mechanisms • Supplementary information

mice and Syrian golden hamsters during SARS-CoV-2 infection. cale bars, 200 µm. (B, C) Percentage of $TUNEL^{+}$ cells per total cells in K18-hACE2 mice and Syrian golden hamsters during SARS-CoV-2 infection based on a 10× high-power field (HPF) (n = 4-5 per dpi). All data are presented as mean ± SEM. *P* values were obtained by one-way analysis of variance; dpi means days post-infection of SARS-CoV-2. Control represents the non-infected animals.

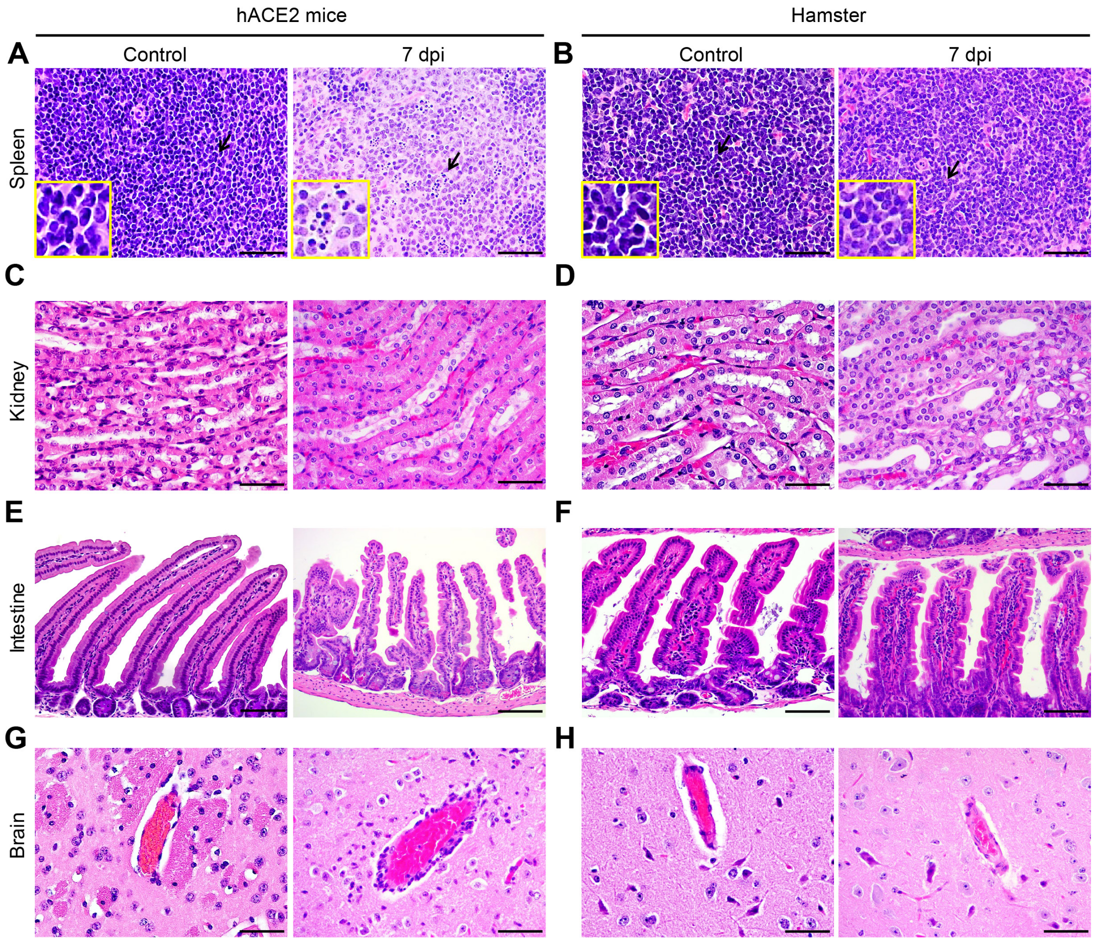

Fig. S2. Pathological features in other organs of SARS-CoV-2-infected K18-hACE2 mice and Syrian golden hamsters. (A, B) Representative hematoxylin and eosin (H&E)- stained images of the spleen in SARS-CoV-2-infected K18-hACE2 mice and Syrian golden hamsters at 7 days post-infection (dpi). Black arrows indicate normal splenocytes or apoptotic cells in the white pulp region shown in the enlarged images in the bottom left corners. Scale bars, 50 µm. **(C, D)** Representative H&E-stained images of the collecting ducts of the kidney in SARS-CoV-2-infected K18-hACE2 mice and Syrian golden hamsters at 7 dpi. Scale bars, 50 µm. **(E, F)** Representative H&E-stained images of the intestine in SARS-CoV-2-infected K18-hACE2 mice and Syrian golden hamsters at 7 dpi. Scale bars, 100 µm. **(G, H)** Representative H&E-stained images of the brain in SARS-CoV-2-infected K18-hACE2 mice and Syrian golden hamsters at 7 dpi. Scale bars, 100 µm. Control represents the non-infected animals.

Disease Models & Mechanisms • Supplementary information

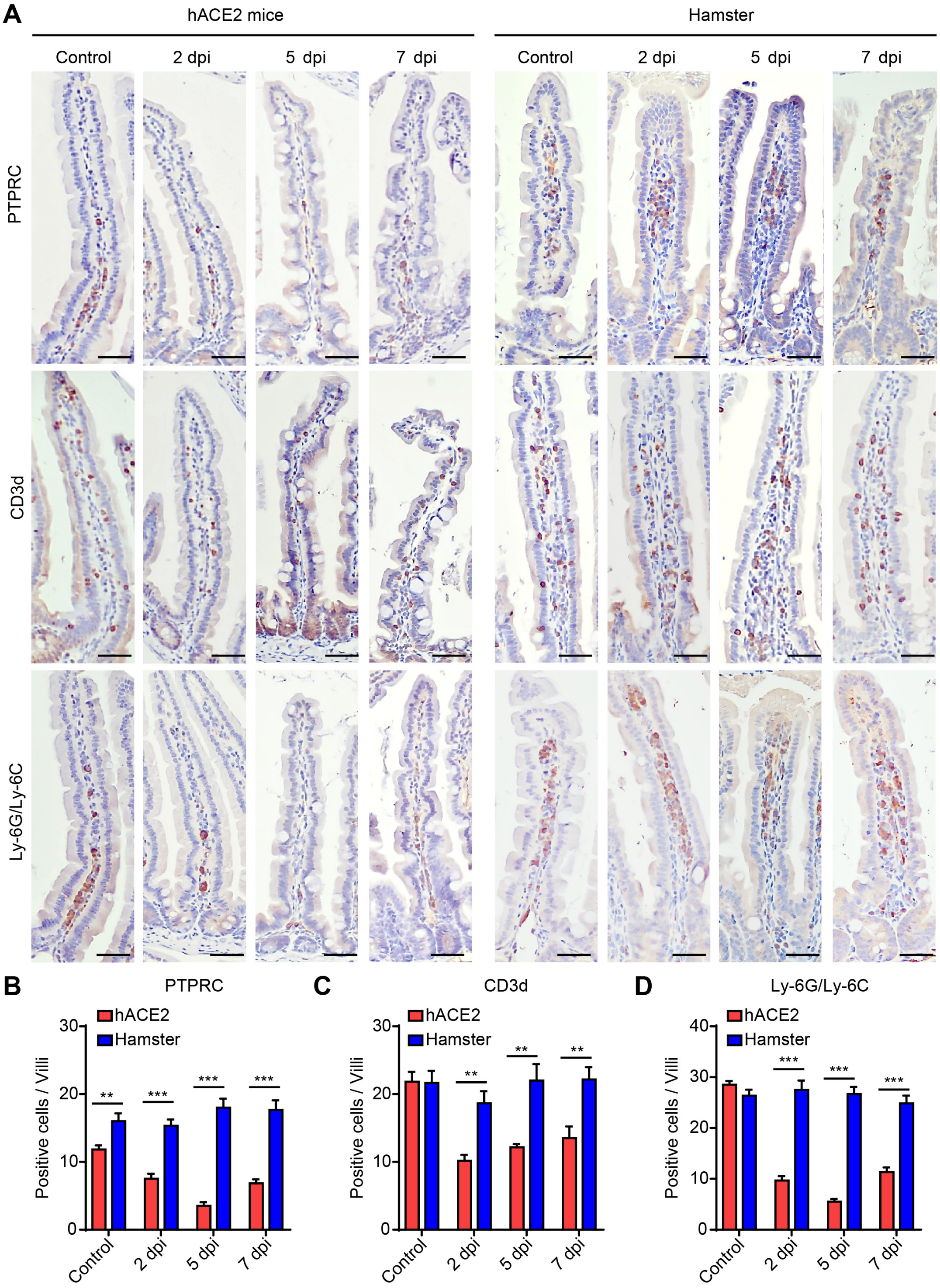

Disease Models & Mechanisms • Supplementary information

Fig. S3. Comparison of intestinal immune subsets in SARS-CoV-2-infected K18- hACE2 mice and Syrian golden hamsters. (A) Representative immunohistochemistry images for PTPRC, CD3d, and Ly-6G/Ly-6C in the intestines of SARS-CoV-2-infected K18-hACE2 mice and Syrian golden hamsters. Scale bars, 50 µm. **(B–D)** Numbers of PTPRC-, CD3d-, and Ly6G/Ly6C-positive cells per single villi, respectively (n = 6 per group). Control represents non-infected animals. *P* values were obtained by two-tailed unpaired Student's *t*- test (**P* < 0.05; ****P* < 0.001). All data are presented as the mean ± SEM. dpi, days post- infection of SARS-CoV-2.

Disease Models & Mechanisms • Supplementary information

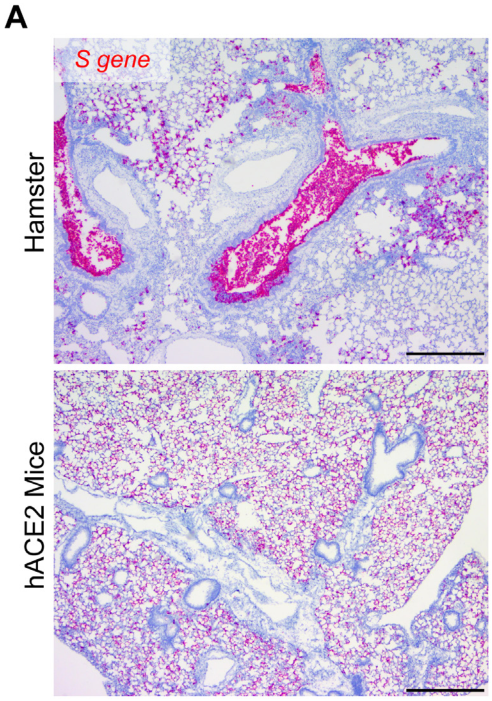

Fig. S4. High-dose SARS-CoV-2 infection in lung. (A) Representative *in situ* hybridization images for *S gene* in lung of 1x10^6 PFU SARS-CoV-2 infected K18-hACE2 mice and Syrian golden hamster at 3 dpi. Scale bars, 500 µm.

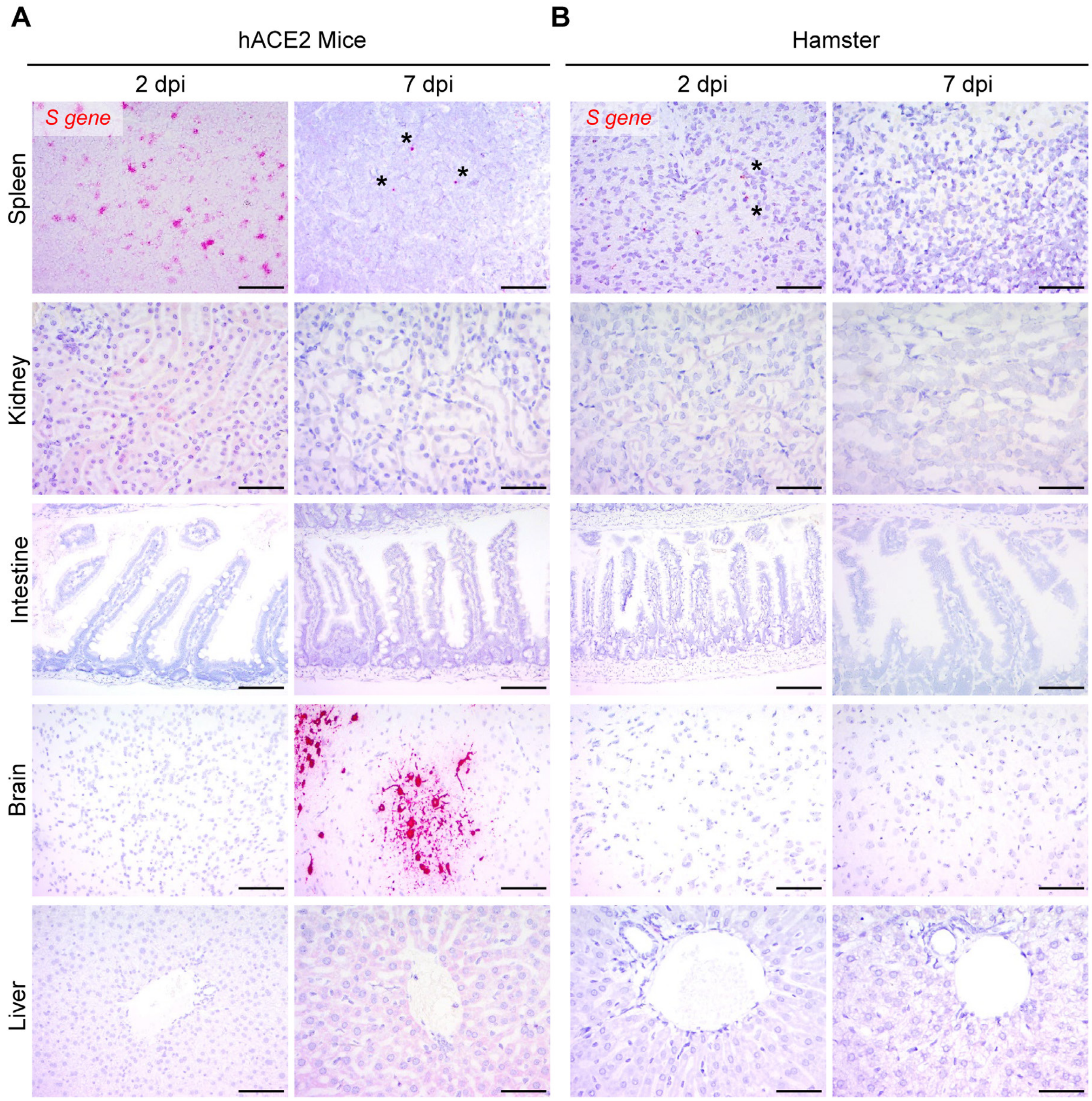

Fig. S5. Systemic infectivity of SARS-CoV-2. (A, B) Representative *in situ* hybridization images for *S* gene in the spleen, kidney, intestine, brain, and liver of SARS-CoV-2-infected K18-hACE2 mice and Syrian golden hamsters at 2 days post-infection (dpi) and 7 dpi. The black asterisk in the spleen indicates *S* gene^{+} splenocytes. Scale bars, 100 µm (intestine and brain); 50 µm (spleen, kidney, and liver).

Disease Models & Mechanisms • Supplementary information

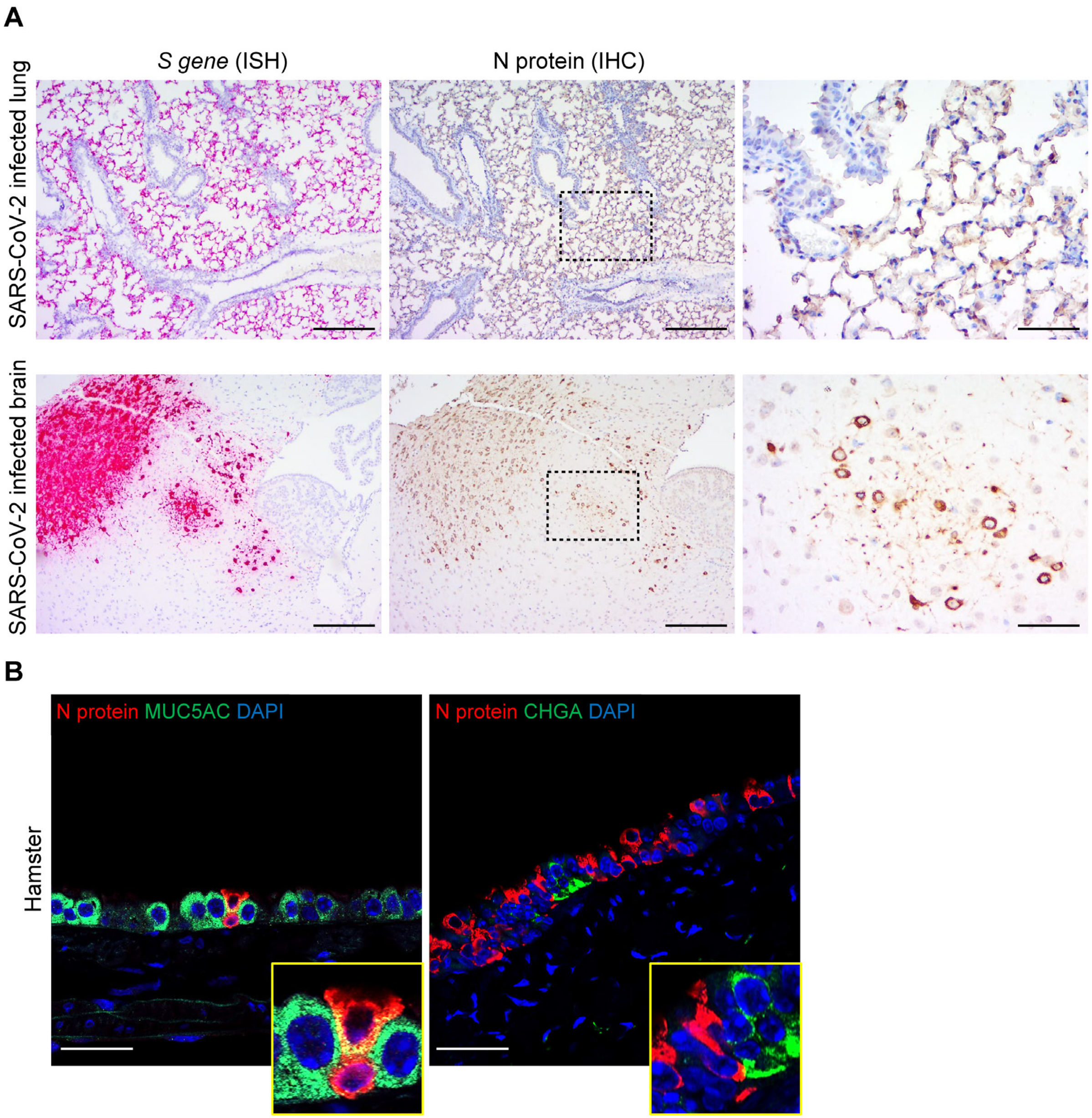

Fig. S6. Comparison of staining patterns of SARS-CoV-2 S gene and N protein. (A) Representative *in situ* hybridization images for *S* gene and immunohistochemistry images for N protein of the lung and the brain in SARS-CoV-2-infected K18-hACE2 mice at 7 days post- infection (dpi). Scale bars, 50 μm (left and middle panels); 100 μm (right panels). **(B)** Representative immunofluorescence images for N protein, MUC5AC (goblet cell marker), CHGA (neuroendocrine cell marker), and DAPI (nuclear marker) in the lungs of Syrian golden hamsters at 2 dpi. Scale bars, 40 μm.

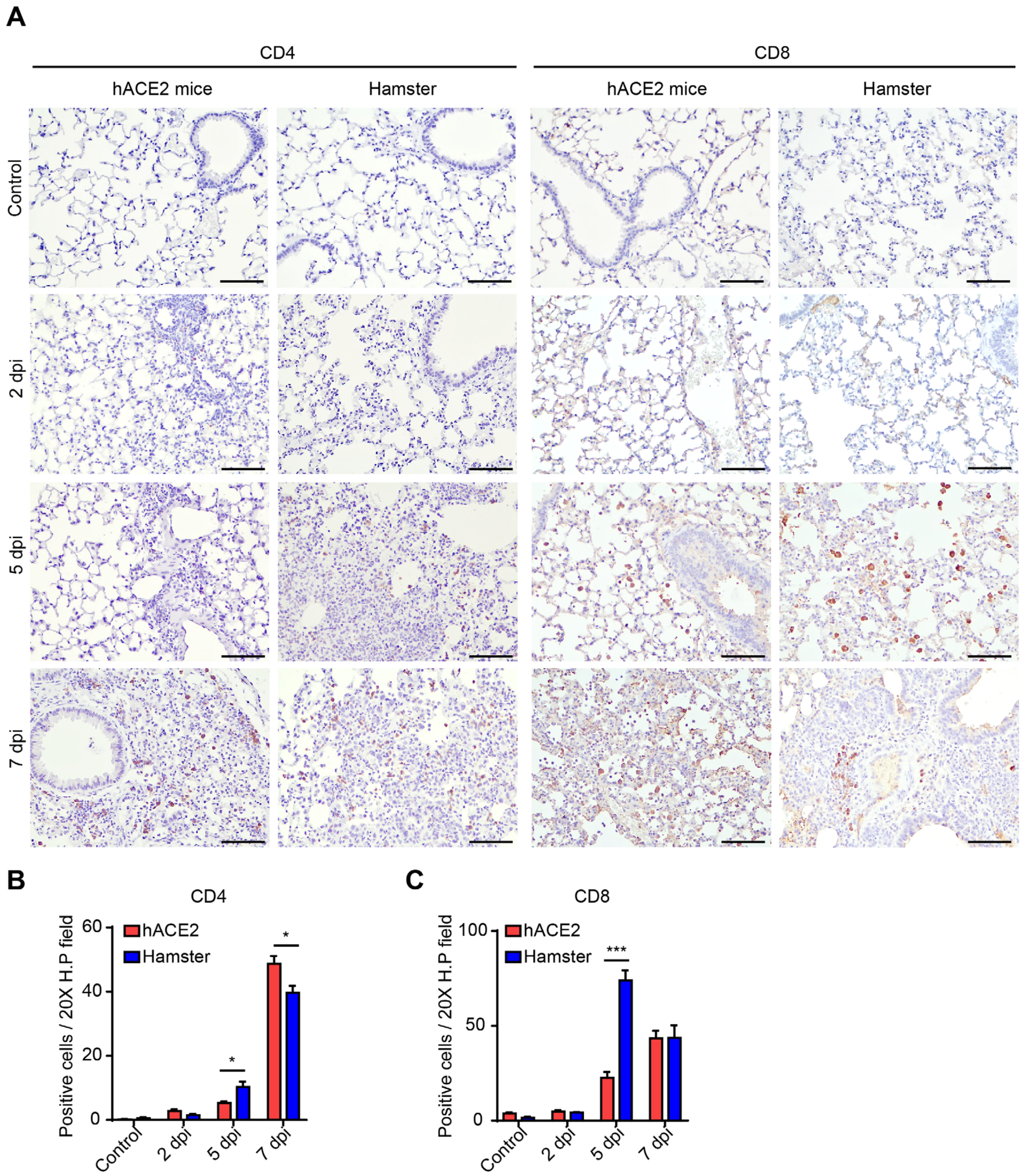

Fig. S7. Comparison of T cell subsets in SARS-CoV-2-infected K18-hACE2 mice and Syrian golden hamsters. (A) Representative immunohistochemistry images for CD4 and CD8 in SARS-CoV-2-infected K18-hACE2 mice and Syrian golden hamsters. Scale bars, 100 µm. **(B–C)** Numbers of CD4- and CD8-positive cells in a 20× high-power field, respectively (n = 6 per group). Control represents non-infected animals. *P* values were obtained by two-tailed unpaired Student's *t*-test (**P* < 0.05; ***P < 0.001). All data are presented as the mean ± SEM. 71 dpi, days post-infection of SARS-CoV-2.

Disease Models & Mechanisms • Supplementary information

Table S1. Complete blood count in SARS-CoV-2-infected K18-hACE2 mice and Syrian golden hamsters. Complete blood count (CBC) in K18-hACE2 mice and Syrian golden hamsters (n = 5 per group). Each data represents the number or percentage of immune subsets. Control represents the corresponding non-infected animal. dpi, days post-infection of SARS- CoV-2.

	hACE2 Mice		Hamster	
	Control	7 dpi	Control	7 dpi
Neutrophil (%)	13.6 (6.3-17.3)	44.1 (34.6-50.8)	20.1 (13.6-36.3)	35.7 (33.5-36.9)
Lymphocyte (%)	79.8 (68.9-86.8)	46.6 (29.3-51.7)	64.5 (47.9-71.6)	46.6 (44.0-49.6)
Monocyte (%)	3.62 (0.7-9.9)	3.3 (0.8-6.5)	7.9 (6.9-9.6)	7.7 (6.4-8.8)
Eosinophil (%)	2.4 (0.8-3.5)	3.9 (2.5-6.4)	4.7 (4.2-5.2)	5.4 (4.2-5.2)
Basophil (%)	0.54 (0.3-0.9)	2.6 (0.9-4.3)	2.7 (2.2-2.9)	4.7 (4.7-6.5)
Red blood cell (10^6/μL)	8.07 (7.3-8.3)	10.9 (9.5-12.5)	8.3 (7.9-8.7)	8.3 (8.2-8.6)
Hemoglobin (g/dL)	12.22 (11.4-14.1)	15.5 (13.6-17.8)	15.5 (15.3-16.0)	15.4 (15.1-15.6)
Hematocrit (%)	39.44 (36.1-44.6)	45.7 (41.2-50.9)	42.8 (42.2-44.4)	42.1 (41.9-42.7)

Disease Models & Mechanisms • Supplementary information
